# Design of targeted primers based on 16S rRNA sequences in meta-transcriptomic datasets and identification of a novel taxonomic group in the Asgard archaea

**DOI:** 10.1186/s12866-020-1707-0

**Published:** 2020-02-03

**Authors:** Ru-Yi Zhang, Bin Zou, Yong-Wei Yan, Che Ok Jeon, Meng Li, Mingwei Cai, Zhe-Xue Quan

**Affiliations:** 1 Ministry of Education Key Laboratory for Biodiversity Science and Ecological Engineering, Institute of Biodiversity Science, School of Life Sciences, Fudan University, Shanghai, China; 2 Laboratory for Marine Fisheries Science and Food Production Processes, Qingdao National Laboratory for Marine Science and Technology, Key Laboratory of Maricultural Organism Disease Control, Ministry of Agriculture and Rural Affairs, Yellow Sea Fisheries Research Institute, Chinese Academy of Fishery Sciences, Qingdao, China; 3 School of Life Sciences, Chung-Ang University, Seoul, South Korea; 4 Shenzhen Key Laboratory of Marine Microbiome Engineering, Institute for Advanced Study, Shenzhen University, Shenzhen, China; 5 Key Laboratory of Optoelectronic Devices and Systems of Ministry of Education and Guangdong Province, College of Optoelectronic Engineering, Shenzhen University, Shenzhen, China

**Keywords:** Meta-transcriptomic datasets, 16S rRNA, Universal primer, Asgard, Taxonomy

## Abstract

**Background:**

Amplification of small subunit (SSU) rRNA genes with universal primers is a common method used to assess microbial populations in various environmental samples. However, owing to limitations in coverage of these universal primers, some microorganisms remain unidentified. The present study aimed to establish a method for amplifying nearly full-length SSU rRNA gene sequences of previously unidentified prokaryotes, using newly designed targeted primers via primer evaluation in meta-transcriptomic datasets.

**Methods:**

Primer binding regions of universal primer 8F/Arch21F for bacteria or archaea were used for primer evaluation of SSU rRNA sequences in meta-transcriptomic datasets. Furthermore, targeted forward primers were designed based on SSU rRNA reads from unclassified groups unmatched with the universal primer 8F/Arch21F, and these primers were used to amplify nearly full-length special SSU rRNA gene sequences along with universal reverse primer 1492R. Similarity and phylogenetic analysis were used to confirm their novel status.

**Results:**

Using this method, we identified unclassified SSU rRNA sequences that were not matched with universal primer 8F and Arch21F. A new group within the Asgard superphylum was amplified by the newly designed specific primer based on these unclassified SSU rRNA sequences by using mudflat samples.

**Conclusion:**

We showed that using specific primers designed based on universal primer evaluation from meta-transcriptomic datasets, identification of novel taxonomic groups from a specific environment is possible.

## Background

Since Leeuwenhoek discovered microorganisms through his microscope, studies have investigated microbial footprints by using various methods. Using traditional culturing methods, various microorganisms have been isolated and studied; however, these methods only encompassed 1% of the microorganisms [1–3]. Although novel microorganisms can be identified by improving the cultivation media, the potential for identifying novel microorganisms remains limited [4]. By fingerprinting small subunit (SSU) ribosomal RNA (rRNA) sequences, Woese classified all life into three domains: Eukarya, Bacteria, and Archaea [5]. This method provided novel avenues for microbial molecular taxonomic classification. In the early 1990s, SSU rRNA genes were evaluated to analyze microbial community structures in environmental samples [6]. Subsequently, amplification of SSU rRNA gene fragments using universal primers, along with next-generation sequencing, has been broadly applied to analyze environmental microbial community structures [7, 8]. However, many microbes have not yet been identified owing to limited coverage of universal primers [9–11]. Therefore, universal primer-independent sequencing library construction methods are needed. Recently, single-cell genome or metagenome sequencing has been applied for identifying microbial dark matter [12–14]. Through deep metagenome sequencing of ultra-small microbes, Anantharaman et al. [14] reported 47 novel candidate phyla, of which 46 were not identified by 16S rRNA gene sequencing with universal primers.

With the application of single cell genome and metagenome sequencing and assembly, the number of phyla in Archaea domain has increased to more than twenty, and these phyla clustered to Euryarchaota and three superphyla—TACK, DPANN, and Asgard [15]. According to the assembled genomes from metagenome sequencing, the Asgard superphylum embraced six phyla: *Lokiarchaeota*, *Thorarchaeota*, *Heimdallarchaeota*, *Odinarchaeota*, *Helarchaeota*, and *Gerdarchaeota* [16–19]. The phylogenetic and comparative genomic analysis showed that Asgard archaea share a common ancestry with eukaryotes and even putatively have eukaryotic signatures [17], initiating a discussion about three or two domains [20–24]. Recently, a Lokiarchaeota-related strain was firstly isolated [25].

However, single-cell genome and metagenome sequencing have shown limited capacity to discover microbial dark matter because massive data are needed for metagenome assembly and binning. Modified meta-transcriptomic methods that were used to enrich SSU rRNA without universal primers [26] provided an opportunity to discover active microbial dark matter from the “rare biosphere” not covered by universal primers.

**Fig. 1:**
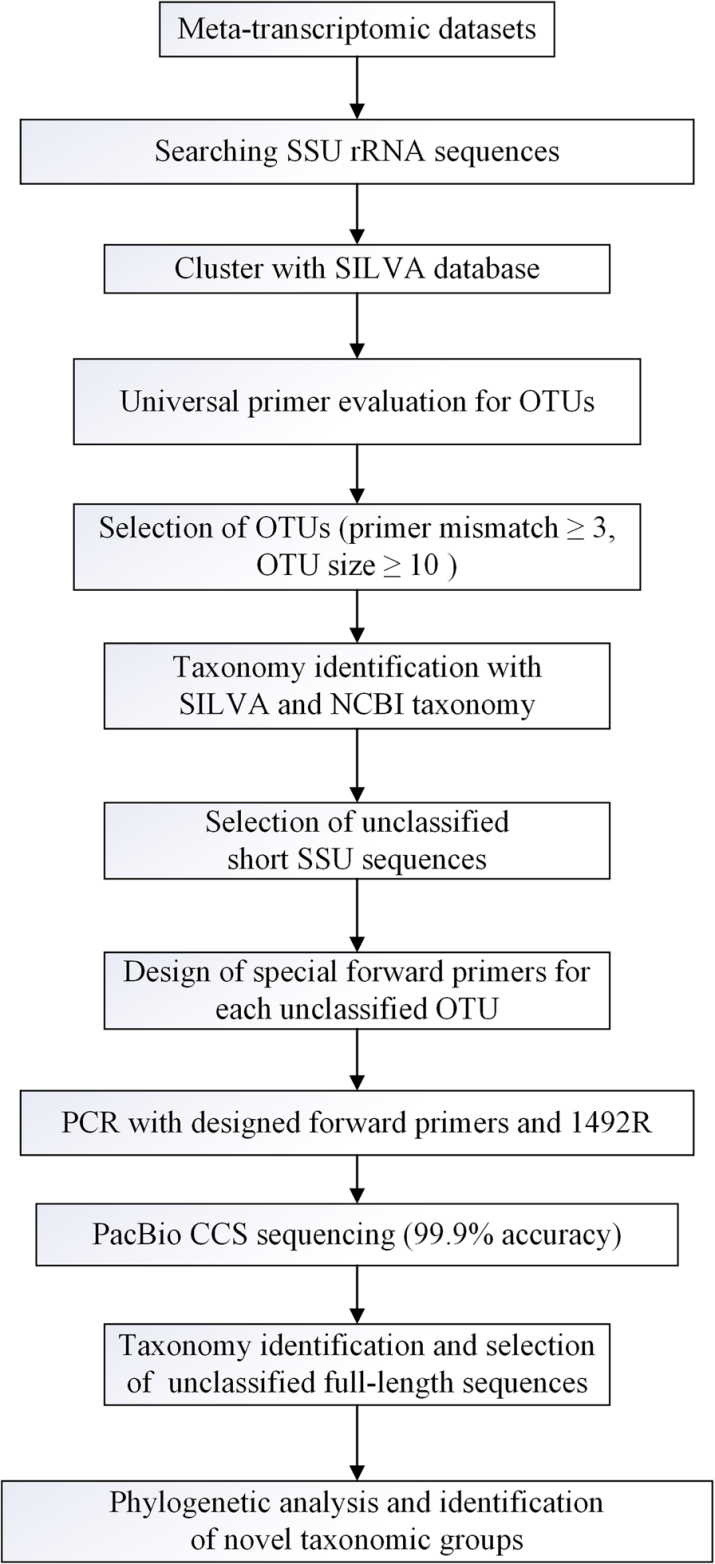
Schematic representation of a pipeline for data mining for novel taxonomic groups

To identify “rare biosphere” microbial dark matter from meta-transcriptomic datasets, we developed a method for designing new primers based on SSU rRNA datasets by using a modified meta-transcriptomic method [26] and amplified nearly full-length 16S rRNA genes of novel taxa by using these newly designed forward primers and universal reverse primer (Fig. 1).

## Results

### Screening of SSU rRNA and primer evaluation

From the meta-transcriptomic data obtained from mudflat sediments, 833,297 SSU rRNA sequences were detected using SSUsearch, accounting for 51.0% of the total meta-transcriptomic sequences. There were 470,813 sequences with complete 8F or Arch21F binding sites. Within these, a total of 454,931 SSU rRNA sequences were completely covered by primers 8F and Arch21F, with no mismatch found; however, the remaining 15,882 SSU rRNA sequences were mismatched (Table 1). If a read contained more than 1 mismatched nucleotide with primer or contained only 1 mismatch but at four nucleotides of the 3′ end of the primer [10], the read was considered as mismatching. Within these mismatched sequences, 11,145 sequences were unclassified at the phylum level. SSU rRNA sequences detected using SSUsearch, combined with SILVA_123 SSURef non-redundant (Nr) sequences, clustered into 23,756 operational taxonomic units (OTUs), with a cutoff of 0.85. The OTUs with sequence number ≥ 10 were retained for analysis, including 1839 bacterial OTUs, 27 archaeal OTUs, and three domain unclassified OTUs. Within the mismatched 16S rRNA sequences, five archaeal and six bacterial OTUs of unclassified phyla were obtained using SILVA_123 (Table 2). In these OTUs, OTU8 and OTU11 were unclassified at the phylum level, and no classified query sequences with identity over 0.8 were found in the NCBI Nr database. For the convenience of description, OTU11 was referred as Type A and OTU8 as Type B, respectively, both of which were not related to chloroplast and mitochondrial sequences according to Metaxa analysis [27].

**Table 1:** Summary of the evaluation of universal primers for meta-transcriptomic data of mudflat sediments [26], using MIPE

Domain	Primer	Incompleteness^a^	Completeness		
			matching^b^	mismatching	
				No. mismatch = 1, 2	No. mismatch ≥3
Bacteria	8F	9603	452,840	12,664	894
Archaea	Arch21F	45	2060	1695	585
Unclassified	Arch21F	187	31	10	34

^a^ Number of reads that only contain incomplete binding sites for primers 8F or Arch21F

^b^ Reads completely matched with the primer or only contain 1 mismatch not at the last four nucleotides of the primer were considered matching; others, mismatching

**Table 2:** Types of mismatches among primer sites meeting the screening criteria

Primer	OTU	Mismatch type	OTU size	SILVA_Tax	NCBI_Tax
8F	OTU1	==G==A====A=========	49	Unclassified	Planctomycete
	OTU2	==G==A====A=========	33	Unclassified	Planctomycete
	OTU3	==G==A====A=========	34	Unclassified	Planctomycete
	OTU4	==G==A====A=C=======	11	Unclassified	Planctomycete
	OTU5	==G==A=====T========	10	Unclassified	Planctomycete
	OTU6	==G==A====A=C=======	10	Unclassified	Planctomycete
Arch21F	OTU7	C==T=============A==	69	Unclassified	Crenoarchaeota
	**OTU8 (Type B)**	**=====T=C=======A====**	**25**	**Unclassified**	**Unclassified**
	OTU9	=A=T=============A==	24	Unclassified	Euryarchaeota
	OTU10	C===A===========T===	10	Unclassified	Euryarchaeota
	**OTU11 (Type A)**	**C==TA===========TA==**	**12**	**Unclassified**	**Unclassified**

Evaluation of universal primers for mudflat meta-transcriptomic SSU rRNA sequences [26]. Screening criteria: the sequences of this OTU are taxonomically unclassified at the domain or/and phylum level, primer-binding regions in each sequence of this OTU are complete and the mismatched bases with the universal primer ≥3, and the sequence number in the OTU ≥10. OTUs identified in unclassified phyla with both SILVA_123 and NCBI BLAST are shown in boldface

### Specific primers for two groups of novel sequences

**Fig. 2:**
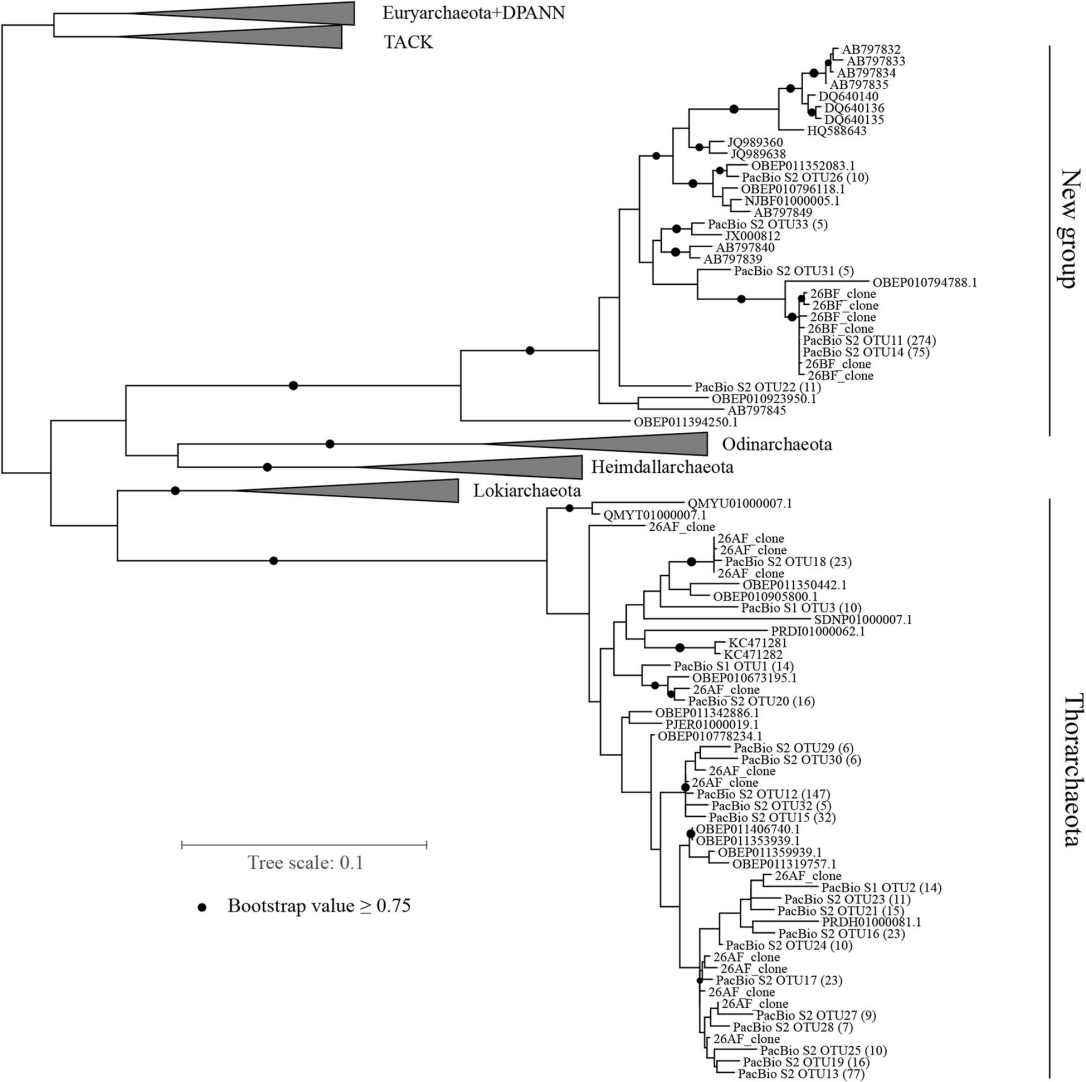
Maximum likelihood (ML) phylogenetic tree for 16S rRNA gene sequences in the Asgard superphylum. Label 27BF refers to 16S rRNA gene clones amplified using primers 27BF and 1492R; Label 26AF, 26AF and modified 1492R. S1 and S2 labels indicate samples S1 and S2. PacBio sequences in this tree are representative operational taxonomic unit (OTU) sequences, and numbers of sequence labels in the parentheses represent the sizes of OTUs, and only OTUs ≥5 are shown herein. Two samples constituted phylum *Thorarchaeota* and a new group. Reference sequences were selected from SILVA_132 database, NCBI nucleotide Nr database, and genome taxonomy database (GTDB). Asgard-related 16S rRNA sequences reported by Karst et al. [28] are also included (labels start with OBEP). The bootstrap support value was set to 1000, and nodes above 0.75 are denoted by black circles. All alignment sequences are > 1200 bp. The scale bar indicates the number of substitutions per site

As shown in Table 3, after performing Primer_BLAST, several specific primers were designed, including three Type A-specific forward primers (targeting OTU11), six Type B-specific forward primers (targeting OTU8), and one degenerate primer covering both groups of sequences. Reverse primer 1492R and its modified version were used to amplify nearly full-length 16S rRNA gene sequences with newly designed specific forward primers from sample S1 (Table 3). The specifications of these primers were confirmed using clone libraries. Although primer specifications were confirmed via Primer BLAST and RDP Probe match, most forward primers mostly amplified non-target microorganisms, except 26AF and 27BF (Table 3). As shown in Fig. 2, these sequences amplified by 26AF and 27BF clustered with reference sequences form two different groups of the Asgard superphylum. To increase the coverage in the Asgard superphylum, primer 26ABF covering Type A and Type B groups was designed, and two different sequences were successfully amplified together from Guangtan mudflat subsurface sediment sample S1. This primer also amplified these two sequences from Guangtan mudflat subsurface sediment sample S2 (Additional file 2: Figure S1).

**Table 3:** Newly designed primers and clone libraries generated using these primers for sample S1

Target	Forward_primer	Reverse_primer	Annealing (°C)	Results of clone library
Type A	21AF: CTCTAGTTGATCCTGCTAGA	1492R-22 W: TACGG(A/T)TACCTTGTTACGACTT	47	Euryarchaeota (26/28) ^b^
	26AF: GG(A/G)CACTGCTATCGGCTT		52	Unclassified (14/14)
	59AF: AAGTCGAACGGACACGCAT		52	- ^a^
Type B	12BF: CGATCCTGACGGAGCCTA	1492R: GGTTACCTTGTTA(C/T)GACTT	45	–
	15BF: ATCCTGACGGAGCCTA		45	–
	16BF: TCCTGACGGAGCCTAC		45	–
	17BF: CCTGACGGAGCCTACT		50	Crenarchaeota (11/13)
	26BF: GCCTACTGCTATCGGATT		45	Thaumarchaeota (8/10)
	27BF: CCTACTGCTATCGGATT		45	Unclassified (8/9)
Type A and Type B	26ABF: G(G/C)(G/A/C)(C/T)ACT GCTATCGG(A/C)TT	1492R: GGTTACCTTGTTA(C/T)GACTT	50	Unclassified (39/100)

^a^ There were no obvious 16S rRNA gene bands after amplification

^b^ Abundance of the majority microorganisms in clone results

### Phylogenetic status of full-length novel 16S rRNA gene sequences

As shown in the phylogenetic tree in Fig. 2, all 13 sequences amplified using 26AF clustered within *Thorarchaeota*, and six sequences amplified with 27BF belonged to the Asgard superphylum, but did not cluster in any known specific phylum, thus forming novel groups with some unclassified reference sequences.

**Fig. 3:**
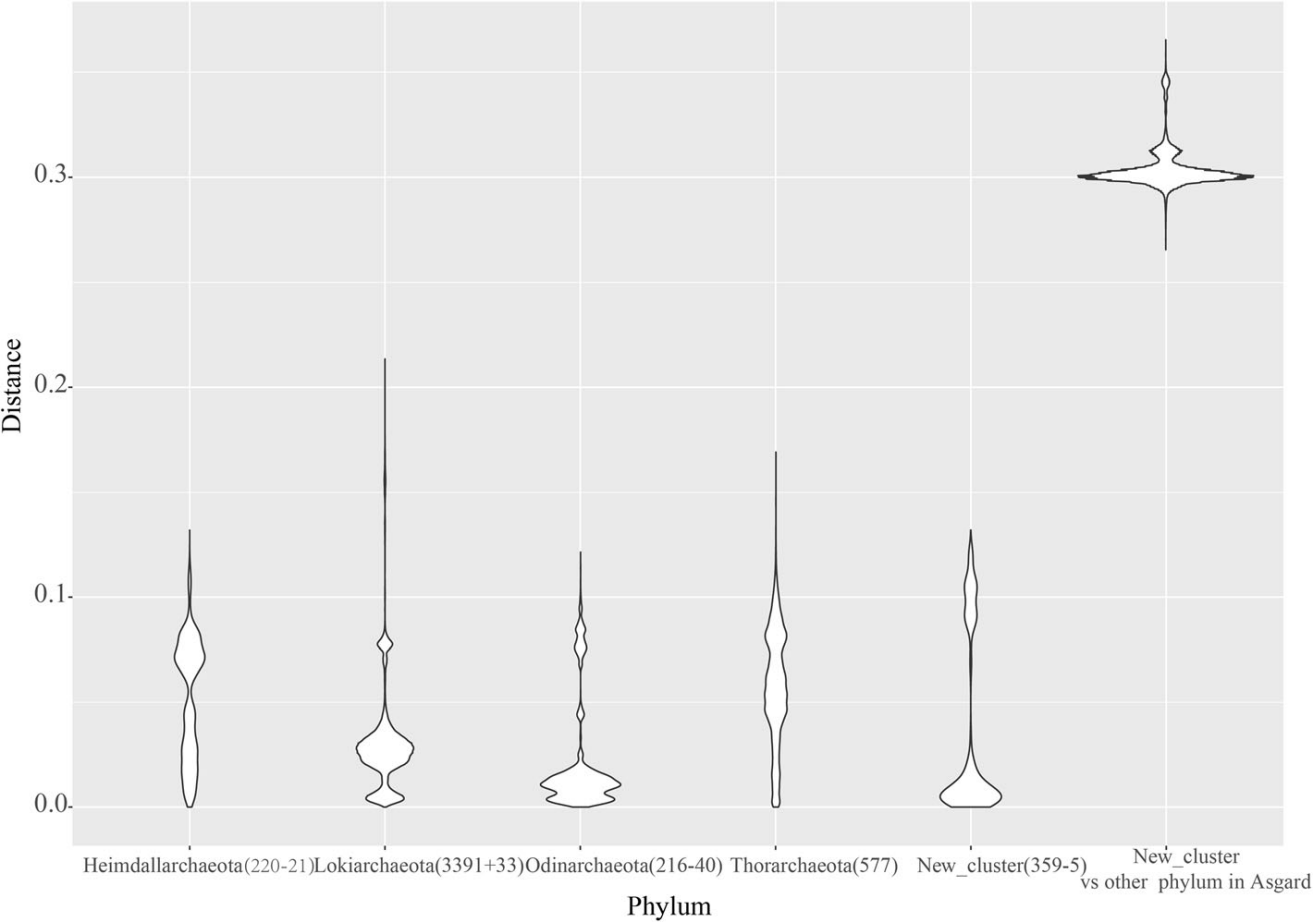
Violin plot for distances in the sequences of Asgard superphylum. All 16S rRNA gene sequences of Asgard superphylum of > 1200 bp in SILVA_132, NCBI, and GTDB were included. Distances between new groups and other phyla, which are related to the new group in the phylogenetic tree, are shown. Numbers in parentheses denote the sequence numbers in SILVA database and the number of sequences added or removed after the re-classification of phylogenetic analysis

To improve the coverage of the novel taxonomic types in the Asgard superphylum, primer 26ABF, encompassing both groups of novel sequences, was used to amplify sample S1. Upon amplification with primers 26ABF and 1492R, 59 unclassified sequences were identified in the Asgard superphylum in the total of 193 clone sequences from three mudflat samples. Through PacBio RS II sequencing, 557 and 1139 clean sequences were obtained after filtering chimeras separately from samples S1 and S2. Among these sequences, 45 sequences in sample S1 and 868 sequences in sample S2 corresponded to the Asgard superphylum. Using a cutoff of 0.97, 10 and 57 OTUs were obtained from samples S1 and S2, respectively. As shown in Fig. 2, 16 reference sequences and 367 sequences obtained by PacBio sequencing in this study clustered together and were separated from the identified phyla in the Asgard superphylum. Other PacBio sequences clustered with phylum of *Thorarchaeota* references. In the distance matrix shown as a violin plot (Fig. 3), the minimum distance between new group sequences and four phyla of the Asgard superphylum was over 0.25. The maximum intra-distance of the new group was 0.12. A local BLAST revealed that the maximum identity for short sequences of Type B with PacBio sequenced full-length 16S rRNA gene sequences of the novel group was from 88 to 93%, and the maximum identity for short sequences of Type A with PacBio sequenced full-length 16S rRNA gene sequences of *Thorarchaeota* was from 99.46 to 100% (Additional file 1: Table S1). This confirmed that Type A and Type B are the same as the respective PacBio-assessed novel full-length 16S rRNA gene sequences, respectively, affiliated with a new group and phylum of *Thorarchaeota*.

## Discussion

The advent of next-generation sequencing has yielded numerous SSU rRNA sequences in datasets potentially amplified using universal primers. However, the mismatches of universal primers potentially result in a PCR bias, thus missing certain microbial types [10, 29]. The read lengths generally obtained via next-generation sequencing for PCR amplifiers are less than 600 base pair (bp), and these relatively short sequences have limitations related to accurate classification and identification with similarity or phylogenetic analysis [30]. To accurately classify microorganisms, full-length 16S rRNA gene sequences are typically amplified using primer 8F or Arch21 with universal primer 1492R [31–34]; however, primer bias remains inevitable [10, 29]. Some studies amplified nearly full-length unclassified 16S rRNA gene environmental sequences with universal reverse primers, and specifically designed forward primers based on pyrosequencing datasets of the hypervariable regions V1 and V2 regions of 16S rRNA amplified using a bacterial universal primer pair [32]. However, the designed special forward primers also have some limitations for the identification of novel taxa because the datasets of hypervariable regions V1 and V2 regions used for primer design were obtained from amplification with a bacterial universal primer set [10].

We previously established an experimental method to analyze the microbial community structure using meta-transcriptomic data. In the meta-transcriptional assay, the 5′ terminus of SSU rRNA was ligated to the adaptor with a tag sequence and reverse-transcribed using a random primer with a tag sequence. Therefore, most SSU rRNA sequences determined with this method contained complete 5′ termini [26]. To amplify full-length novel microbial SSU rRNA sequences, the 5′ termini of SSU rRNA sequences from this dataset were used for primer evaluation with Microbiota metagenome Primer Explorer (MIPE) software [35]. Using MIPE software, we aligned the SSU rRNA sequences, extracted the primer (8F or Arch21F) binding sites, and obtained mismatch information regarding these primer sites with universal primers. As shown in Table 1, about 54.6% of the SSU rRNA sequences completely encompassed the primer region of primers 8F and Arch21F, 50.0% of sequences with a complete Arch21F binding site were mismatched by Arch21F, and 2.8% of sequences with an 8F binding site were mismatched by 8F. Within the mismatched sequences, 70.2% sequences were unclassified at the phylum level. Thus, this method can be used to amplify nearly full-length 16S rRNA genes of novel taxonomic groups with targeted primers designed from 16S rRNA sequences of meta-transcriptomic datasets, and Archaea may be the main target for discovering microbial dark matter.

In this study, OTUs meeting the criteria were focused, and two types of novel sequences (Type A and Type B) uncovered by the universal forward primer Arch21F were detected. To identify novel taxonomic groups matched to these novel sequences, we designed separate targeted forward primers 26AF and 27BF based on 16S rRNA short sequences of Type A and Type B separately which were used to amplify full-length novel SSU rRNA gene sequences, and these clone sequences were affiliated with the Asgard superphylum (Fig. 2). To determine more diverse full-length 16S rRNA gene sequences in the Asgard superphylum, a new degenerate forward primer 26ABF covering Type A and Type B were designed. Upon sequencing fragments amplified with 26ABF and 1492R with PacBio, we obtained diverse sequences belonging to the Asgard superphylum (Fig. 2). Although sequences related to those of Type A and Type B (Table 2) only occupied approximately 1/20,000 of the original SSU rRNA sequences in the meta-transcriptomic datasets [26], one-third of the full-length SSU rRNA gene sequences via PCR amplification with targeted primers designed solely based on sequences in Type A (12 sequences) and Type B (25 sequences) were affiliated with the Asgard superphylum. Microbial taxa with a relative abundance below 0.01% [36] were defined as rare biospheres, which normally may be discarded when performing microbial community analysis. Although rare-biosphere microorganisms occupy the minority of samples, they provide a broad reservoir of ecological function and resiliency, displaying specific and sometimes unique ecology and biogeography that can differ substantially from that of more abundant microorganisms [37]. Thus, it is important to find and identitfy these organisms. Designing targeted primers using 16S rRNA sequences from meta-transcriptomic datasets potentially improves the resolution of novel microorganism analysis and increases the probability of identifying rare biospheres.

The distance between the new group and sequences of other phyla in the Asgard superphylum was over 0.25, averaging approximately 0.3 (Fig. 3). Considering 0.75 as the proposed threshold sequence identity at the phylum level [29], this new group should be a candidate phylum of the Asgard superphylum. References DQ640135, DQ640136, and DQ640140 were amplified using 4F (5′-TCCGGTTGATCCTGCC(A/G)G-3′) and 1492R [38]. However, primer 4F contained three mismatches against 16S rRNA sequences in Type B from meta-transcriptomic datasets. Most other amplified reference sequences in this candidate phylum were amplified by primer Arch21. Primer evaluation by using MIPE revealed mismatches between Arch21 and new group sequences (Table 2). Therefore, these reference sequences may have resulted from non-targeted amplification with mismatched primers. In addition to the novel group, numerous 16S rRNA gene sequences affiliated with phylum of *Thorarchaeota* were obtained. Karst et al. [28] also found Asgard-related full-length 16S rRNA sequences by using a combination of primer independent poly(A)-tailing reverse transcription and synthetic long-read sequencing methods. In their sequencing results, we also found some sequences affiliated with the new group and *Thorarchaeota*. In the group of the phylum *Thorarchaeota*, the reference sequences KC471281 and KC471282 were derived from 179 clone sequences of the marine sediment 16S rRNA poly-A-tailed reverse transcript product [39]. No nearly full-length Thorarchaeotal 16S rRNA gene sequences (> 1200 bp) have been amplified with primers before this study. According to the NCBI taxonomy database, 75 metagenome assemblies were identified as the members of the Asgard superphylum. However, because of the massive data volume, short read length, skewed species abundance, and high similarity of 16S rRNA genes between different taxon, only 18 assemblies contained the nearly full-length 16S rRNA gene. Only one assembly’s 16S rRNA gene was related to a novel group, and 29 assemblies were related to *Thorarchaeota*, with six showing approximately full-length 16S rRNA genes. Limited information regarding the 16S rRNA gene sequence may prevent the accurate identification of 16S rRNA gene sequences during microbial population analysis. Recently, Sriram et al. [40] suspected the reality of Asgard archaea. Their analyses revealed that Asgard metagenomic assembled genomes are binning artifacts, assembled from environments where up to 90% of DNA originates from dead cells. However, the Asgard archaea we found originate from meta-transcriptomic datasets, thus confirming the existence of active microbes in the Asgard superphylum.

Similar to previous studies [38, 41, 42], *Thorarchaeota* and the novel group-related sequences obtained in this study originated from sedimental environments based on the design of specific primers according to 16S rRNA sequences in meta-transcriptomic datasets, indicating that the two groups prefer anoxic or anaerobic environments. This agrees with studies of anaerobic-related genes in *Thorarchaeota* genomes [41, 43].

Recently, the Asgard superphylum has received increased attention with respect to evolutionary biology. In the genome of microbes in the Asgard superphylum, markers have been identified that were previously considered to be present only in eukaryotic microbes [17, 21, 41]. This finding led to the speculation on the origin of eukaryotes and whether microbes should be divided into two or three domains [20–24]. Cai et al. [42] confirmed that Asgard archaea are diverse and ubiquitous and proposed five previously unknown subgroups for the Asgard superphylum by clustering the publicly available Asgard archaeal 16S rRNA gene sequences. Through meta-transcriptomic analysis reported previously [26] and performed herein, combined with primer evaluation and specific primer design, we amplified mudflat samples and enriched the 16S rRNA gene diversity of the Asgard superphylum from mudflat samples. Identification of a novel candidate phylum in the Asgard superphylum might enable further studies of the evolutionary relationship between Archaea and Eukarya and improve the current understanding of the two-domain or three-domain theory.

## Conclusions

By evaluating universal primer sites in the meta-transcriptomic datasets, we identified a candidate novel phylum in the Asgard superphylum that was not covered by the universal primer Arch21F. To our knowledge, this is the first study to amplify 16S rRNA gene sequences of the phylum *Thorarchaeota*. Combinatorial application of fluorescence in situ hybridization with probes specific to this novel group and fluorescence-activated cell sorting and genomic analysis of this novel group might provide insights into the evolutionary relationship between Archaea and Eukarya. Using widespread meta-transcriptomic and metagenomic data, specific primers may be designed by universal primer evaluation for samples obtained from diverse environments to further identify previously unreported microbes.

## Methods

### Screening of SSU rRNA sequences and primer evaluation

SSUsearch [44] was used to identify read names of SSURef rRNA sequences by screening 12 meta-transcriptomic datasets of previously reported mudflat samples obtained through Illumina Miseq PE300 sequencing [26]. Original SSU rRNA sequences were obtained using Mothur’s (v.1.33.3) command *list.seqs* and *get.seqs* [45], since SSU rRNA sequences obtained by SSUsearch did not contain 5′ ends, which are important for evaluating universal forward primers 8F/Arc21F.

Identified SSU rRNA sequences were combined with SILVA_123^1^ SSU Nr sequences [46] and clustering in accordance with 85% similarity using Usearch 8.0 [47]. First, combined sequences were sorted using the *sortbylength* command. Next, the sorted sequences were clustered using the *cluster_fast* command. SSU rRNA sequences and representative sequences of OTUs were submitted to MIPE^2^ to evaluate the universal primers [35]. The primers used for primer evaluation were bacterial universal primer 8F (5′-AGAGTTTGAT(C/T)(A/C)TGGCTCAG-3′) [48] and archaeal universal primer Arch21F (5′-TTCCGGTTGATCCTGCCGGA-3′) [49]. Based on the results of MIPE primer evaluation, potential novel OTUs were screened in accordance with the following criteria: the sequences of this OTU are taxonomically unclassified at the domain or/and phylum level, primer-binding regions in each sequence of this OTU are complete and the mismatched bases with the universal primer are ≥3, and the sequence number in the OTU is ≥10. Next, two OTUs with phylum unclassified were blasted against the NCBI Nr database. Metaxa [27] was finally used to verify that these two unclassified OTUs were not mitochondrial or chloroplast sequences.

### Specific primers designed for novel sequences and screening

Specific forward primers with a *T*_*m*_ of 45–52 °C and length of 16–20 nucleotides (nt) were designed based on new groups of meta-transcriptomic SSU rRNA sequences by using Primer_BLAST [50]. These forward primers were localized in the first 100 nt of novel meta-transcriptomic SSU rRNA sequences. All forward primers were evaluated using RDP probe match [51] to prevent non-target amplification. Universal primer 1492R (5′-GGTTACCTTGTTA(C/T)GACTT-3′) [52, 53] covering eukaryotic, bacterial, and archaeal sequences was used as the reverse primer. Phylogenetic analysis of clone sequences for these specific primers revealed that these two sequence groups clustered within the Asgard superphylum (Fig. 2); accordingly, a degenerate forward primer 26ABF (see in Table 3) covering the sequences of both novel groups was designed to amplify more diverse sequences in the Asgard superphylum.

### Experiments and data processing

Sediment samples used in this study were collected from Dongtan in Chongming Island, Shanghai, China (121°57′E, 31°33′N), on October 7, 2015, using short core samplers. Sample collection and preservation methods were described previously [26]. Genomic DNA was extracted from preserved sediment samples using the DNeasy Power Soil Kit (QIAGEN, Hilden, Germany) in accordance with manufacturer’s protocol and stored at − 20 °C for subsequent experiments. The DNA concentration was quantified using a Qubit 2.0 Fluorometer (Thermo Fisher Scientific, Waltham, MA, USA). Specially designed forward primers and universal reverse primer 1492R were used to amplify sample S1 (one sediment sample of 15–40-cm depth) to confirm primer specificity and efficiency. Amplifications were performed using Ex Taq MIX (TAKARA, Shiga, Japan) by using the primers shown in Table 3 as follows: 95 °C for 5 min, 35 cycles of denaturation at 94 °C for 45 s, annealing at the temperature shown in Table 3 for 30 s, extension at 72 °C for 90 s, and a final extension at 72 °C for 10 min. The target bands were extracted and used to construct clone libraries. Clone fragments were sequenced via 3730 sequencing and assembled. Full-length 16S rRNA gene sequences were submitted to SILVA for in silico molecular taxonomy^3^ (Min. identity with query sequence, 0.8; reject sequences with an identity below 0.7; other parameters, default). Primers specific to the unclassified sequences were further used to amplify same-depth samples S2. Subsequently, amplified fragments were sequenced via 3730 sequencing. Sanger sequencing results were analyzed as described above.

PCR products for which primers (26ABF) showed high specification and coverage were sequenced using the PacBio RS II system according to the standard manufacturer’s conditions. Raw sequences were filtered for a minimum of 3 passes and a minimum predicted accuracy of 99.9%. Circular consensus sequencing fasta files were obtained from the fastq file using the *fastq.info* command (pacbio = T) in Mothur. Primers and barcodes were trimmed using the *trim.seqs* command (checkorient = T, pdiffs = 1, maxambig = 0, maxhomop = 8, qaverage = 60). Clean data were obtained after Qiime chimera filtering (−n 18) [54]. Clean sequences were classified and aligned using SILVA online Alignment^3^, Classification and Tree Service, and sequences of unclassified phyla clustering in accordance with a cutoff of 0.97 using Usearch8 [47]. Representative sequences of OTUs with more than five sequences and unclassified clone sequences were aligned with reference sequences from the NCBI nucleotide non-redundant database, SILVA_132^4^ database, and Genome taxonomy database (GTDB) [55] using SINA 1.4.0 [56] and then filtered using the *filter.seqs* command in Mothur. Trimmed sequences were used to construct a phylogenetic tree to verify their novel taxonomic status via MEGA5.0, using the maximum likelihood (ML) method with a bootstrap test of 1000 replicates [57]. The length of trimmed sequences was over 1200 bp. Phylogenetic trees were analyzed, and figures were generated using iTOL^5^ [58]. To further confirm the novel taxonomic features or novel candidate phylum status of these sequences, Asgard reference sequences obtained from SILVA_132, NCBI, and GTDB were aligned with novel sequences using SINA 1.4.0 and then filtered using the *filter.seqs* command in Mothur. Aligned sequences were trimmed to 1200 bp and submitted to Mothur to determine the distance matrix using the *dist.seqs* command. Their taxonomy was reconfirmed by phylogenetic analysis. The distance matrix was visualized using the ggplot2 violin plot. The relatedness of short sequences in the two novel groups against full-length novel 16S rRNA gene sequences was also determined using a local BLAST.

## Supplementary information

**Additional file 1: Table S1.** Results of local blastn. Novel short-length 16S rRNA as query sequences and PacBio novel full-length 16S rRNA genes as subject sequences.

**Additional file 2: Figure S1.** Phylogenetic tree constructed based on 16S rRNA gene sequences of the degenerate primer amplicon clone. The tree shows the diversity of the 16S rRNA gene clone sequences amplified using degenerate primer 26ABF. This primer was applied in three sediment samples S1, S2 (depth, 15–40 cm). Other 16S rRNA gene reference sequences were retrieved from SILVA_132, NCBI, and GTDB databases. The phylogenetic tree was reconstructed based on 16S rRNA gene sequences derived from the Asgard superphylum via the maximum likelihood method, using MEGA5.0. All 16S rRNA gene sequences are > 1200 bp. The scale bar indicates the number of substitutions per site. The bootstrap support value was set to 1000, and nodes above 0.75 are denoted with black circles.

## Data Availability

Meta-transcriptomic data were reported in our previous study “Microbial Communities and Diversities in Mudflat Sediments Analyzed Using a Modified Metatranscriptomic Method” and have been deposited in the United States National Center for Biotechnology Information (NCBI) with BioProject PRJNA400589. The 16S rRNA gene sequencing data of Asgard candidate phylum and phylum *Thorarchaeota* amplified in this study have been deposited in NCBI GenBank under the accession numbers MN444044-MN444134 and in NODE^6^ under project ID OEP000692.

## Abbreviations

bp: Base pair
GTDB: Genome taxonomy database
MIPE: Microbiota metagenome Primer Explorer
ML: Maximum likelihood
Nr: Non-redundant
nt: Nucleotides
OTUs: Operational taxonomic units
SSU rRNA: Small subunit ribosomal RNA

## Footnotes

1 https://www.arb-silva.de/no_cache/download/archive/release_123/Exports

2 https://github.com/zoubinok/MIPE

3 https://www.arb-silva.de/aligner

4 https://www.arb-silva.de/no_cache/download/archive/release_132/Exports

5 https://itol.embl.de
