# Supplementary figures and images for: Design of targeted primers based on 16S rRNA sequences in meta-transcriptomic datasets and identification of a novel taxonomic group in the Asgard archaea
Source: BMC Microbiol. 2020 Feb 3;20:25. doi: 10.1186/s12866-020-1707-0 (PMC6998087; doi:10.1186/s12866-020-1707-0)

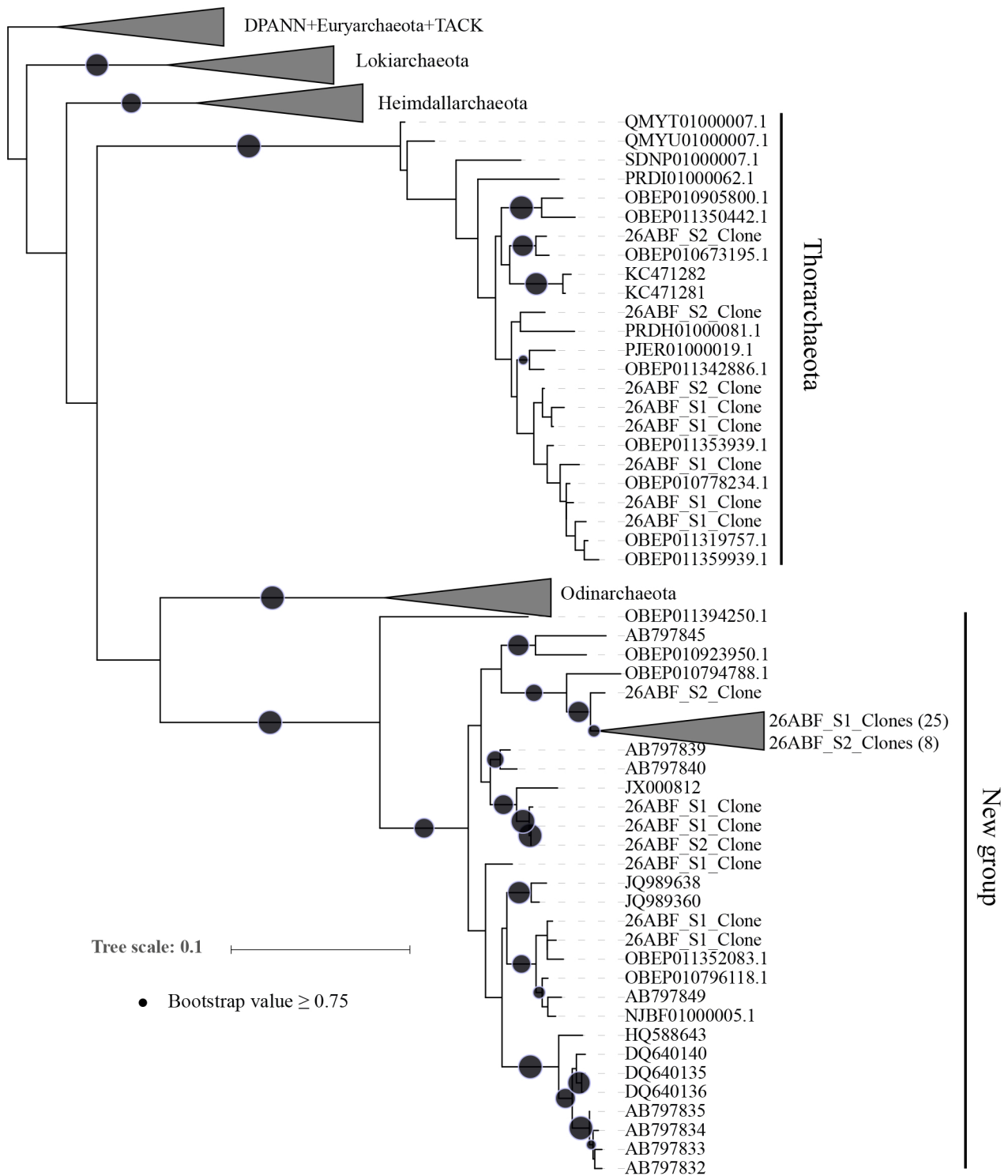

Supplement: Supplementary file 2 — Additional file 2: Figure S1. Phylogenetic tree constructed based on 16S rRNA gene sequences of the degenerate primer amplicon clone. The tree shows the diversity of the 16S rRNA gene clone sequences amplified using degenerate primer 26ABF. This primer was applied in three sediment samples S1, S2 (depth, 15–40 cm). Other 16S rRNA gene reference sequences were retrieved from SILVA_132, NCBI, and GTDB databases. The phylogenetic tree was reconstructed based on 16S rRNA gene sequences derived from the Asgard superphylum via the maximum likelihood method, using MEGA5.0. All 16S rRNA gene sequences are > 1200 bp. The scale bar indicates the number of substitutions per site. The bootstrap support value was set to 1000, and nodes above 0.75 are denoted with black circles. [file 12866_2020_1707_MOESM2_ESM.pdf]
